# Fractal Dimensions of *In Vitro* Tumor Cell Proliferation

**DOI:** 10.1155/2015/698760

**Published:** 2015-03-25

**Authors:** George I. Lambrou, Apostolos Zaravinos

**Affiliations:** ^1^1st Department of Pediatrics, University of Athens, Choremeio Research Laboratory, Thivon & Levadeias, 11527 Athens, Greece; ^2^Division of Clinical Immunology and Transfusion Medicine, Department of Laboratory Medicine, Karolinska Institute, 171 77 Stockholm, Sweden

## Abstract

Biological systems are characterized by their potential for dynamic adaptation. One of the challenges for systems biology approaches is their contribution towards the understanding of the dynamics of a growing cell population. Conceptualizing these dynamics in tumor models could help us understand the steps leading to the initiation of the disease and its progression. *In vitro* models are useful in answering this question by providing information over the spatiotemporal nature of such dynamics. In the present work, we used physical quantities such as growth rate, velocity, and acceleration for the cellular proliferation and identified the fractal structures in tumor cell proliferation dynamics. We provide evidence that the rate of cellular proliferation is of nonlinear nature and exhibits oscillatory behavior. We also calculated the fractal dimensions of our cellular system. Our results show that the temporal transitions from one state to the other also follow nonlinear dynamics. Furthermore, we calculated self-similarity in cellular proliferation, providing the basis for further investigation in this topic. Such systems biology approaches are very useful in understanding the nature of cellular proliferation and growth. From a clinical point of view, our results may be applicable not only to primary tumors but also to tumor metastases.

## 1. Introduction

Population dynamics and population genetics provide a well-developed mathematical theory of evolution [1, 2] and many of these models and techniques have been applied to cancer. Cells growing under normal conditions can manifest proliferation dynamics of nonlinear nature [3, 4]. This nonlinear behavior has also been demonstrated in cells being under the influence of drugs or other environmental factors [5]. Any further knowledge regarding the mechanisms underlying cellular proliferation is of major importance and even the smallest indication towards a certain direction could enable us to discover novel differences in the mechanisms that distinguish healthy from diseased cells.

Genes manifest several patterns of differential expression in cancer [6, 7]. Gene expression is highly correlated to the chromosome level and gene expression data can be simulated using polynomial functions [8–10]. Gene expression has also been suggested to take place discretely and not continuously (i.e., in quanta) [11, 12]. It has also been reported to follow oscillatory patterns, thus complicating things even more regarding the rate of cellular proliferation, be it either growth acceleration or deceleration [13, 14]. In terms of growth rate, this means that cells cannot simply transit from one state to the other. If the hypothesis of oscillatory modulation of gene expression is correct, a much more complicated regulatory pattern should be required by a cell in order to be able to change its state, as a result of environmental stimuli. Biological systems are dynamic systems and it is critical to know how to determine a cell's present state from its previous one. This knowledge can have a vast number of applications, from cancer to insect population control. However, discovering the laws that underlie biological systems is a tedious work. On the one hand it is not easy to model such systems due to their high complexity and, on the other hand, biological dynamical systems possess significant capabilities of adaptation. We tested this hypothesis, adding specific modifications to our previously published experimental setup [15]. Although several studies have dealt with the complex dynamic behavior of animal populations [16–19], little is known regarding the dynamics of tumor cell proliferation [3] and even less is known regarding the state of proliferation dynamics until cells reach an adequate number for the tumor to be diagnosed.

Data regarding the dynamic nature of a tumor can only be collected after it has been diagnosed. Usually, this is too late for the patient, since all the critical steps for the progression of the tumor have already taken place. Therefore,* in vitro* systems provide an excellent opportunity to study effects that are impossible to be measured* in vivo*. Most importantly,* in vitro* systems can be studied in the long term. This is required in order to reach conclusions regarding nonlinearity and chaotic behavior of a cellular system. Since primary cell cultures are short-lived when untransformed (15–20 days), the only way to apply such measurements is to use already established cell lines. For this reason, we developed a modeling approach in order to simulate the* in vivo* conditions, as best as possible. The nature of proliferation dynamics can give insight into the way that not only cells proliferate, but they also differentiate.

In the present study we used systems biology approaches and focused on the dynamics of* in vitro* cellular systems, using three central nervous system (CNS) tumor and a T-cell acute lymphoblastic leukemia (T-ALL) cell lines. These cells provide an excellent substrate for modeling proliferation dynamics, as previously shown [15]. The questions that we posed were as follows. If certain physical measures, including cellular proliferation, are observed at the phenotypical level of the cells, how can they be translated at the molecular or genomic level? If the proliferation rate of a cellular population increases, does this mean that there are genes being transcribed faster than others and/or at a faster rate than usual? We aimed to test the hypothesis that cell proliferation is of nonlinear nature and manifests self-similarity patterns with its subsequent applications. Our results highlight the fact that tumor cells manifest self-similarities in their proliferation potential. This implies that the trajectory of a cellular population can be predicted and it could be a factor determining metastasis.

## 2. Materials and Methods

### 2.1. Cell Cultures

The TE671 (cerebellar rhabdomyosarcoma) [20–23], A172 (glioblastoma) [24], 1321N1 (astrocytoma) [25, 26], and CCRF-CEM (T-ALL) [27–31] cell lines were used as the model, obtained from the European Collection of Cell Cultures (ECACC, UK).

### 2.2. Cell Cultures Conditions

Cells were grown in DMEM and RPMI-1640 medium, 15% FBS, and 0.1x streptomycin/penicillin at 37°C, 5% CO_2_, and ~100% humidity. Cells were cultured in 12-well plates and 75 cm^2^ flasks in total medium volume of 2 mL and 25 mL, respectively. Cells were seeded at initial concentrations of 20 cells/*μ*L~200 cells/*μ*L for the CCRFCEM cells and 30, 60, 120, 240, 480, and 960 total cells populations were fed at regular intervals thereafter. Medium changes took place by centrifugation at 1000 rpm for 10 min, the supernatant was discarded, and the remaining cells were re-diluted in 25 mL media and were allowed to grow. Measurements were taken every 12 hours for a total of >500 hours. Cells were passaged at regular intervals by removing old media and adding fresh. Cells were not trypsinized and were allowed to grow up to the point of reaching confluence of 80–90%. This practically removed the dead cells from the system and the remaining cells were allowed to grow again in fresh medium. This allowed modelling of the growth of a tumor (CNS tumors or leukemia) in a space with finite capacity. Removal of cells modelled the circulation that removes dead cells from a particular position in the organism.

### 2.3. Measurements, Experimental Setup, and Model

The CCRF-CEM cells grow in suspension and can therefore provide an excellent model of avascular growth. In addition, the following assumptions were considered for cellular proliferation: (a) extracellular signal transduction takes place autocrinaly; (b) the cellular distribution at the time of seeding and thereafter is considered to be uniform; and (c) nutrient supply was considered to be stable since cells were fed at regular time intervals. All measurements were performed in triplicate. Wolfrom et al. counted the cell population at the end of a time period varying from 5 to 7 days [3]. At the end of this period, cells were trypsinized, measured, and then seeded at an initial concentration of 10^5^ cells per flask. In our study, prior to every measurement, flasks were gently shaken in order to assure that the sample taken consisted of a representative, equally distributed population size. For the cellular growth dynamics study, cells were assayed at least every 48 h and the media renewed every 3–5 days. For the measurements, 200 *μ*L from each flask was measured on an automatic hematology analyzer (CellTaq-*α*, Nihon Kohden). In addition, for the adherent cells, each plate was supplemented with 10% alamarBlue, a nontoxic dye that turns from blue to red due to its oxidation in the mitochondria.

### 2.4. Mathematical Computations

We used a one-dimensional representation based on the assumption that the present state of our system is dependent upon the previous one. So, our system is better described by the logistic equation, as

(1) $$\begin{array}{c} f\left(x_{n+1}\right)=kx_{n}\left(1-x_{n}\right) \end{array}$$

and with respect to time

(2) $$\begin{array}{c} {\dot{x}}_{n}=kx_{n}\left(1-x_{n}\right) \end{array}$$

(the* logistic differential equation*). Both equations belong to the family of logistic equations of the form

(3) $$\begin{array}{c} f\left(x\right)=kx\left(1-x\right), \end{array}$$

where *k* is the proliferation constant. For the analysis of the data we utilized phase-space and return maps and used the geometrical representation, as previously proposed [3]. To find the fractal dimensions of the measured variables, we calculated two fractal variables: *N* and *R*. *N* represents the number of “squares” needed for a fractal shape to be completed and their respective “square size” *R*. By definition, if the first derivative of *d*ln⁡*N*/*d*ln⁡*R* remains constant for a space of *R*, this is the fractal dimension of the shape, in the present case of the cell proliferation trajectory. All mathematical computations were performed in the MATLAB computing environment.

## 3. Results

**Figure 1:**
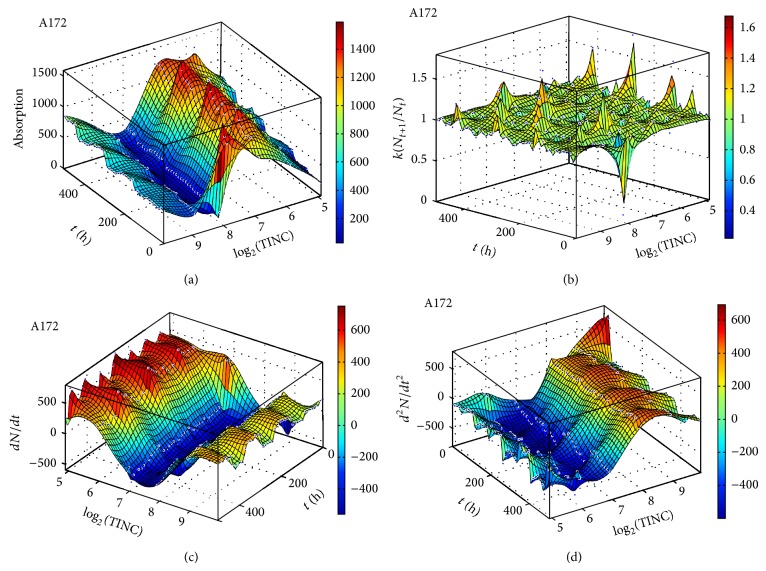
Graphical representation of time-series experiments for the A172 (glioblastoma) cells. Factors presented include cell proliferation (a), rate of growth (*N*_*t*+1_/*N*_*t*_) (b), the speed of growth (*dN*/*dt*) (c), and the acceleration of proliferation (*d* ^2^ *N*/*dt* ^2^) (d). TINC: total initial number of cells.

**Figure 2:**
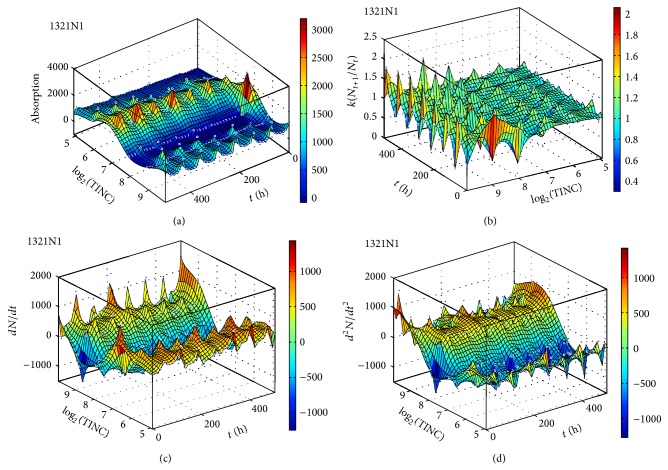
Graphical representation of time-series experiments for the 1321N1 (astrocytoma) cells. Factors presented include cell proliferation (a), rate of growth (*N*_*t*+1_/*N*_*t*_) (b), the speed of growth (*dN*/*dt*) (c), and the acceleration of proliferation (*d* ^2^ *N*/*dt* ^2^) (d). TINC: total initial number of cells.

**Figure 3:**
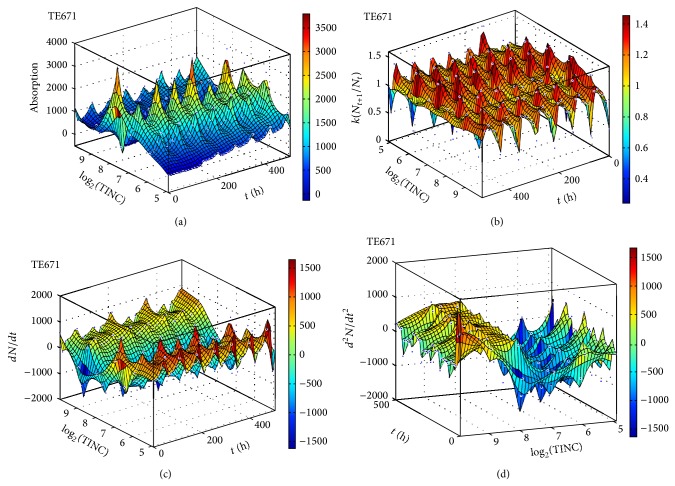
Graphical representation of time-series experiments for the TE671 (cerebellar rhabdomyosarcoma) cells. Factors presented include cell proliferation (a), rate of growth (*N*_*t*+1_/*N*_*t*_) (b), the speed of growth (*dN*/*dt*) (c), and the acceleration of proliferation (*d* ^2^ *N*/*dt* ^2^) (d). TINC: total initial number of cells.

**Figure 4:**
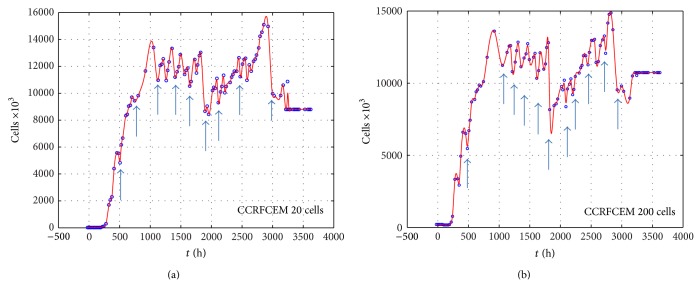
Graphical representation of time-series experiments for the CCRCEM (T-cell acute lymphoblastic leukemia) cells. Factors presented include cell proliferation for 20 cells initial population (a) and 200 cells initial population (b).

**Figure 5:**
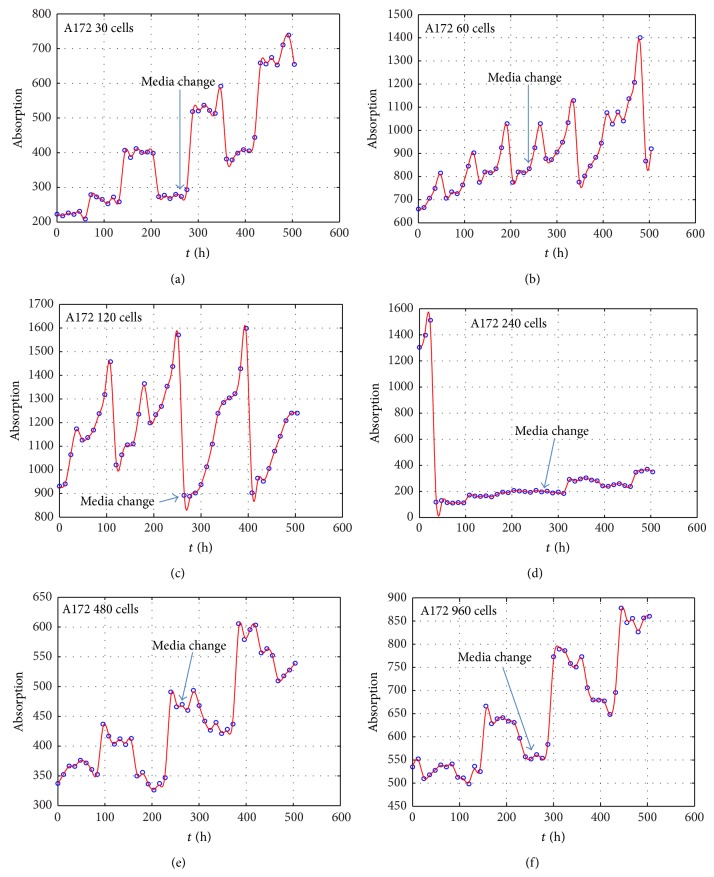
Graphical representation of time-series experiments for the A172 (glioblastoma) cells. Factors presented include the cell proliferation measurements as photometric absorption for different initial cells populations: 30 cells (a), 60 cells (b), 120 cells (c), 240 cells (d), 480 cells (e), and 960 cells (f).

**Figure 6:**
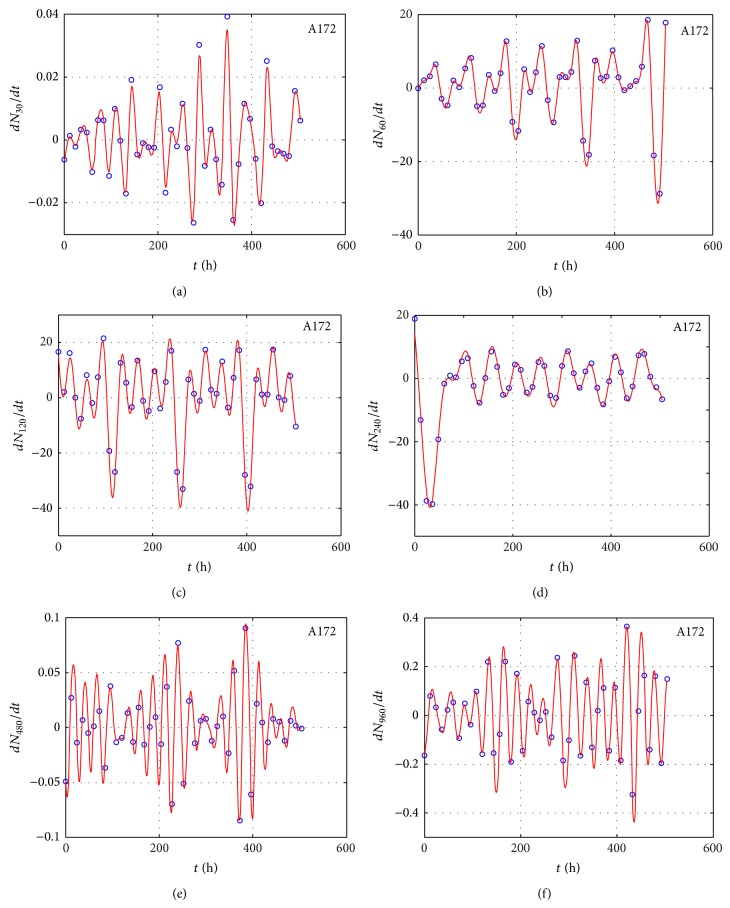
Graphical representation of time-series experiments for the A172 (glioblastoma) cells. Factors presented include the *dN*/*dt* for different initial cells populations: 30 cells (a), 60 cells (b), 120 cells (c), 240 cells (d), 480 cells (e), and 960 cells (f).

**Figure 7:**
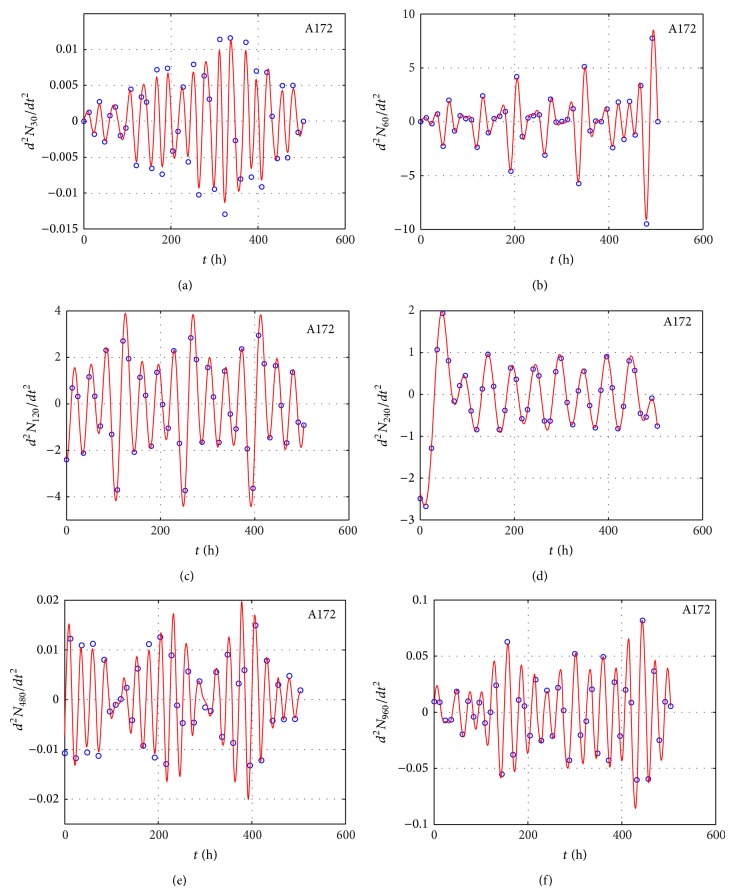
Graphical representation of time-series experiments for the A172 (glioblastoma) cells. Factors presented include the *d* ^2^ *N*/*dt* ^2^ for different initial cells populations: 30 cells (a), 60 cells (b), 120 cells (c), 240 cells (d), 480 cells (e), and 960 cells (f).

**Figure 8:**
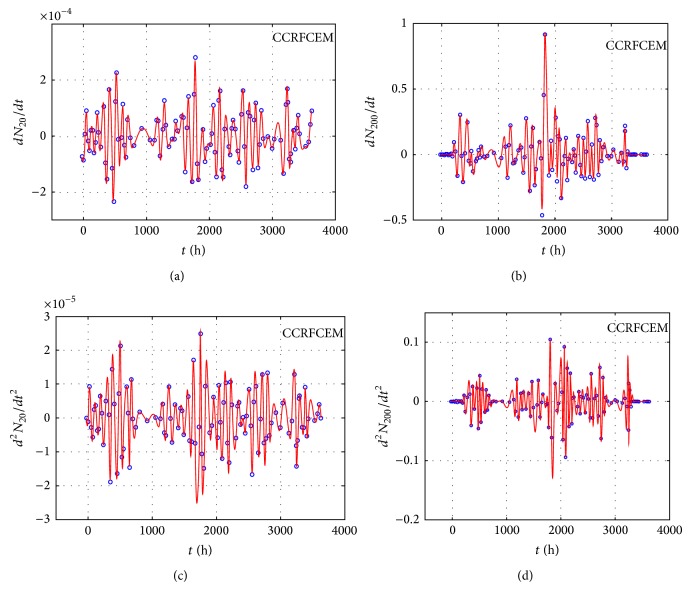
Graphical representation of time-series experiments for the CCRFCEM (T-cell acute lymphoblastic leukemia) cells. Factors presented include the *dN*/*dt* for different initial cells populations: 20 cells (a), 200 cells (b), the *d* ^2^ *N*/*dt* ^2^ for the same populations 20 cells (c), and 200 cells (d).

**Figure 9:**
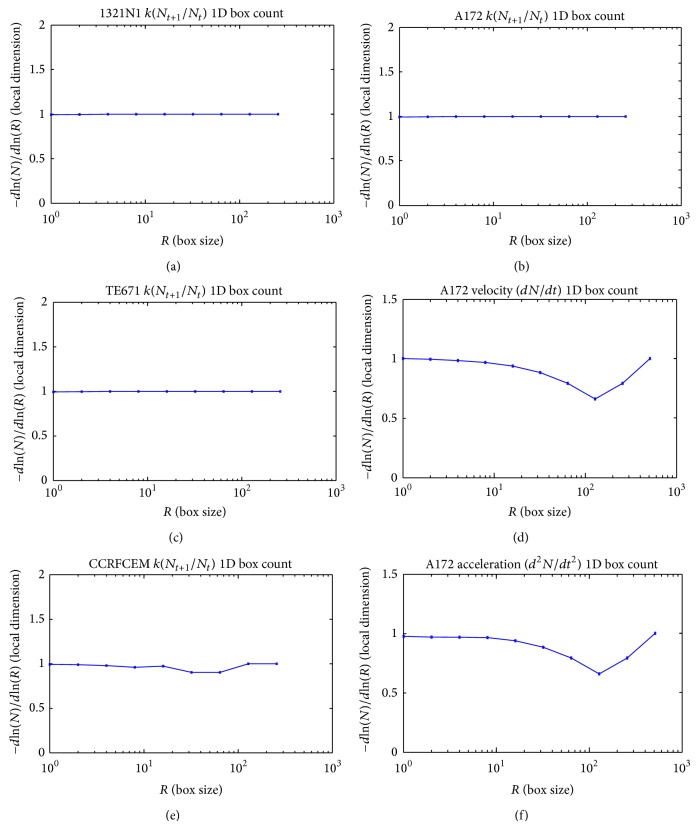
Graphical representation of self-similarity calculations for the 1321N1 cells with respect to rate of proliferation (a), for the A172 cells with respect to rate of proliferation (b), for the TE671 cells with respect to the rate of proliferation (c), for the A172 cells with respect to the velocity of cell growth (d), for the CCRFCEM cells with respect to the rate of proliferation (e), and for the A172 cells with respect to the acceleration of cell growth.

We measured the proliferation of the three CNS tumor cells and the CCRF-CEM cells* in vitro*. Due to the large amount of data, the proliferation results are presented in three-dimensional graphs. The time-series proliferation results in the A172, 1321N1, and TE671 cells revealed that proliferation follows an oscillatory pattern (Figures 1–3). The rate of growth [*N*(*t* + 1)/*Nt*] appeared to manifest the most stable oscillatory pattern, among all measurements that we performed (Figures 1(b), 2(b), and 3(b)). In order to resolve more the patterns of oscillation, we present the proliferative pattern for the CCRF-CEM cells, which resembles that of adherent cells (Figure 4). As a representative resolution, the proliferation dynamics of the A172 cells (Figure 5), characteristic for all adherent cell lines, clearly revealed an oscillatory pattern in cellular growth velocity (Figure 6) and acceleration (Figure 7), respectively. It appears that cells do not proliferate in a linear pattern; rather they oscillate while adapting to the environmental conditions. Apart from testing this in adherent cells, we also applied our question to cells growing in suspension. Of major interest, these cells also exhibited similar dynamics (Figure 8). Therefore, our results support that different cell types manifest similar proliferation patterns, suggesting that a similar self-similarity pattern exists among different cellular types. In order to investigate self-similarity, it was necessary to show that cell proliferation factors follow some form of repetition. In systems biology, when the first derivative *d*ln⁡*N*/*d*ln⁡*R* remains constant in a space *R*, it is a hint of self-similarity. Interestingly, the rate of proliferation was equal to 1 for all cell types, while for the growth velocity and acceleration for the A172 cells it was equal to 0.80888 (Figure 9). In order to conceive the meaning of those numbers, two shapes with the same self-similarity measures are mentioned: the* Cantor sets* (*d*ln⁡*N*/*d*ln⁡*R* = 1) and the* Apollonian Gasket* (self-similarity value = 0.8). Our results confirmed two interesting points: (1) cell growth factors follow oscillatory dynamics (of nonlinear nature) and (2) different cell types followed similar dynamics of growth, irrespective of whether they grow as adherent or suspension cells, hinting towards a common mechanism of cellular proliferation.

## 4. Discussion

In the present work we identified nonlinear factors of cellular proliferation, in three CNS tumor cell lines and one leukemic cell line. Since our results show that cell growth is of nonlinear nature, we propose an initial theoretical framework for the analysis of such phenomena and for future considerations. This knowledge could be useful in treating tumors, since by understanding the mechanisms of cellular proliferation we could interpret the factors that determine the progression of the disease and/or metastasis. Biological systems are extremely complicated and manifest non-linear/chaotic phenomena. Among others [15, 32], we strongly support that the maturity of biological sciences can be achieved through their integration with other disciplines, such as those of mathematics and physics. This integration will enable the research community to give generalized models for phenomena such as the non-linear nature of cellular growth.

We have previously described the chaotic patterns of the leukemic cell line that we used [15]. These patterns were shown by the orbits/trajectories of proliferation and the* Lyapunov* exponents (one of the criteria of chaos existence). Such an example is the understanding of cellular proliferation in which we attempted to contribute with our hints. Here, we show that that different cell types follow similar dynamics with respect to proliferation, however their dynamics follow different trajectories. This difference arises from the probable reason that it is possible that all trajectories can be described by the same function, yet with different constants. Another question arising is that when cellular populations are measured with respect to time, they would give natural numbers corresponding to the exact or approximate number of cells present in the system. At the same time, all other physical variables calculated return oscillations. The question posed, concerns the role of gene expression in controlling cellular proliferation. A possible explanation is that cells follow oscillatory dynamics exactly the same way gene expression does [11].

Another issue in tumor proliferation dynamics that remains unanswered is the conditions at the time of the disease onset. The only knowledge we have thus far, concerning tumors, originates from the time of clinical presentation, at diagnosis. Practically, we have complete lack of knowledge from the time of tumor initiation to the time of tumor presentation. In that sense, the understanding of the proliferation dynamics of tumor cells is critical since it could provide insight into the understanding of tumor initiation. Supposing we could describe the dynamics of cellular proliferation in a formal, mathematical form, we would be able not only to predict the time zero (the starting point) but also to understand the mechanics of this progression. Additionally, if we suppose that such a formal description could be applied to several tumor types, we could conclude to a general rule of tumor proliferation. To the best of our knowledge, there are no previous works dealing with this subject and posing these questions.

The implications of the understanding of proliferation dynamics are immense. To date, we do not have a sufficient theory that could allow us understand and predict cellular growth. This is easy to prove if we ask a simple question: given the population of cells today can we predict the population after 24 hours? The answer is no, since the only way we can do it is by approximation and this can be done only statistically. In other words, if we could predict the future cellular population based on the past population, this could lead us to the starting point of cellular proliferation, information that would be of extreme importance in cancer biology.

Concluding, in the present work we aimed to set a framework for the detection of global patterns in cellular proliferation. Considering the fact that our knowledge in tumor biology comes only from the clinical presentation of the disease, the discovery of global models of tumor progression and proliferation could provide more insight in tumor biology and be used for therapeutic or prognostic purposes. Future work should focus on the investigation of the rates of cellular death in the same proliferation models and, most importantly, expand this model to gene expression for the same proliferation models, thus moving from phenotype to the genotype.
